# Crystal Structure of the Dengue Virus Methyltransferase Bound to a 5′-Capped Octameric RNA

**DOI:** 10.1371/journal.pone.0012836

**Published:** 2010-09-17

**Authors:** Li Jian Yap, Dahai Luo, Ka Yan Chung, Siew Pheng Lim, Christophe Bodenreider, Christian Noble, Pei-Yong Shi, Julien Lescar

**Affiliations:** 1 School of Biological Sciences, Nanyang Technological University, Singapore, Singapore; 2 Novartis Institute for Tropical Diseases, Singapore, Singapore; 3 AFMB UMR6098 CNRS, Marseille, France; Yale University, United States of America

## Abstract

The N-terminal domain of the flavivirus NS5 protein functions as a methyltransferase (MTase). It sequentially methylates the N7 and 2′-*O* positions of the viral RNA cap structure (GpppA→^7me^GpppA→^7me^GpppA_2′-*O*-me_). The same NS5 domain could also have a guanylyltransferase activity (GTP+ppA-RNA→GpppA). The mechanism by which this protein domain catalyzes these three distinct functions is currently unknown. Here we report the crystallographic structure of DENV-3 MTase in complex with a 5′-capped RNA octamer (G_ppp_AGAACCUG) at a resolution of 2.9 Å. Two RNA octamers arranged as kissing loops are encircled by four MTase monomers around a 2-fold non-crystallography symmetry axis. Only two of the four monomers make direct contact with the 5′ end of RNA. The RNA structure is stabilised by the formation of several intra and intermolecular base stacking and non-canonical base pairs. The structure may represent the product of guanylylation of the viral genome prior to the subsequent methylation events that require repositioning of the RNA substrate to reach to the methyl-donor sites. The crystal structure provides a structural explanation for the observed trans-complementation of MTases with different methylation defects.

## Introduction

Dengue virus (DENV) belongs to the genus *Flaviviruses* and is the arthropod-borne etiologic agent of dengue fever, dengue haemorrhagic fever and dengue shock syndrome. Other medically important flaviviruses include West Nile virus (WNV), Japanese encephalitis virus (JEV) and Yellow fever virus (YFV). Currently, there is neither a vaccine nor antiviral therapy licensed for the prevention or treatment of dengue. The flavivirus genome consists of a 11 kb positive strand RNA segment with a cap 1 structure (5′-^7me^G_ppp_A_2′-*O*-me_-RNA-3′), where the guanosine and adenosine are methylated at the N7 position of the base and 2′-*O* position of the sugar respectively. The cap structure is important for RNA stability, binding to ribosomes and efficient translation [1], [2], [3]. The viral RNA consists of 5′- and 3′-untranslated regions (UTR) harbouring several evolutionary-conserved RNA tertiary structures that play important roles in viral RNA replication. Both the 5′ (G_ppp_AG) and 3′ (CU_OH-3′_) sequences are strictly conserved across the flavivirus genomes [4]. A single open reading frame is translated into a long polyprotein precursor which is processed into three structural proteins and seven nonstructural (NS) proteins that replicate RNA [5]. The NS5 protein is a 103 kDa protein consisting of an N-terminal S-adenosyl-L-methionine (SAM)-dependent methyltransferase (MTase) [6], [7] and a C-terminal RNA-dependent RNA polymerase (RdRp) [8], [9], [10], [11]. The RdRp domain synthesizes the RNA replicative intermediates and additional copies of the (+) strand viral RNA that are packaged into nascent virions [12], [13], [14].

The current model for the formation of the RNA cap 1 structure assumes four sequential steps: (1) the 5′-γ-phosphate of the nascent RNA is removed by the RNA triphosphatase activity of the viral protein NS3 [15], [16], [17]. (2) the RNA 5′-diphosphate end is capped by guanosine monophosphate (GMP) through a 5′ to 5′ phosphodiester bond linkage, a step catalyzed by a guanyltransferase (GTase) that might be the NS5 protein itself [18], [19], [20]. (3) The NS5 MTase transfers a methyl group to the N7 position of the guanine moiety forming a cap 0 structure (^7me^G_ppp_A-RNA) [7], [21]. (4) The NS5 MTase catalyzes methylation at the ribose 2′-*O* position of adenosine resulting in formation of the cap 1 structure (^7me^G_ppp_A_2′-*O*-me_-RNA) [6], [22]. After each methylation event, SAM is converted to the by-product S-adenosyl-L-homocysteine (SAH). Three-dimensional structures of several flaviviral MTases bound to SAH, with either GTP or cap analogues, have delineated the co-factor and one cap- binding sites which lie about 16 Å apart. These structures also revealed a positively-charged putative binding site for RNA substrates [6], [21]. Using a scintillation proximity assay, we previously measured the activity of DENV 2′-O MTase with an oligomeric capped RNA substrate: GpppAGAACCUG [23]. This RNA substrate binds to the enzyme with high affinity thus allowing a rapid and sensitive detection of its MTase enzymatic activities.

While structural information about the SAM and GTP binding site with short capped RNA abound, there is a need to study the structure of a flaviviral MTase bound to longer 5′-capped RNA chains, given the requirement for a minimal number of nucleotides for proper MTase activity [24]. To start addressing the molecular basis for the interactions between the flavivirus MTase and longer capped RNA substrates, we solved the X-ray crystal structure of DENV-3 MTase in complex with a 5′-capped octameric RNA at 2.9 Å resolution.

## Results

### Structure determination

**Figure 1:**
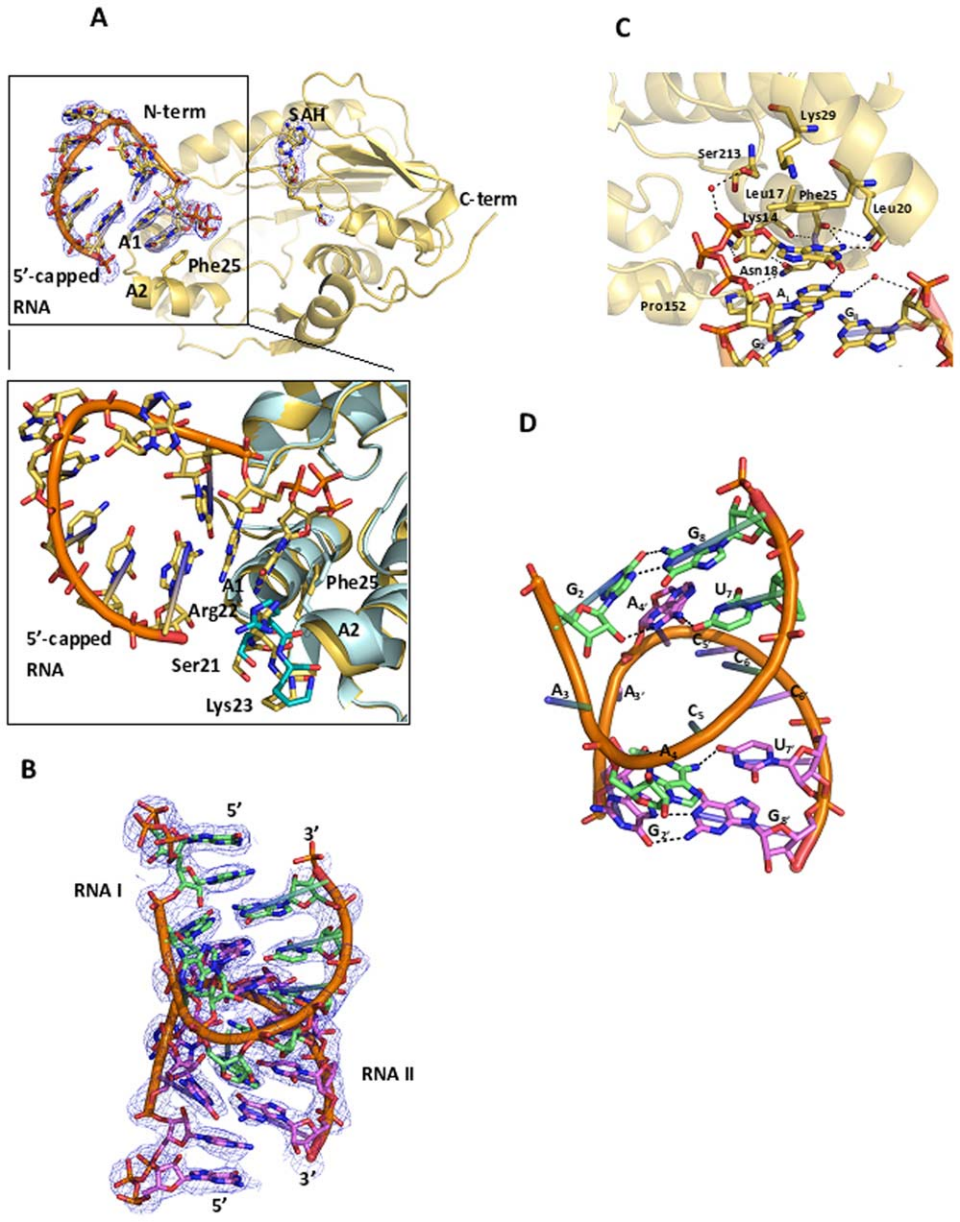
Interactions of the 5′-capped RNA with the MTase. (**A**) Cartoon representation of one MTase monomer with bound RNA octamer and SAH. The F_o_-F_c_ omit electron density map (where both the RNA chain and the SAH have been omitted from the phase calculation) is contoured at a level of 2.5 σ. The SAH co-product, 5′-capped RNA and residue Phe25 are shown as sticks. The inset shows a comparison between RNA bound (yellow) and free (cyan) MTase monomer, highlighting the conformational changes occurring upon RNA binding (see text) (**B**) Structure of the RNA dimer as observed in the MTase-5′-capped RNA complex. The 2F_o_-F_c_ electron density map is contoured at 1.5 σ. (C) Cartoon representation of G_0ppp_A_1_-RNA interacting with residues at the GTP binding site. Water molecules are represented by red spheres. Dotted lines represent hydrogen bonds. Protein residue numbers and bases are also given. Prime numbers indicate RNA bases related by the 2-fold ncs. (D) Cartoon representation of the two RNA chains present in the asu, G_2_A_3_A_4_C_5_C_6_U_7_G_8_ (carbon atoms in green) and G_2′_A_3′_A_4′_C_5′_C_6′_U_7′_G_8′_ (RNA strand related by 2-fold ncs axis; carbon atoms in magenta) Eight non-canonical base-pairs are observed. The A_3_ and A_3′_ moieties of the RNA flip out of from their respective RNA chain.

**Figure 2:**
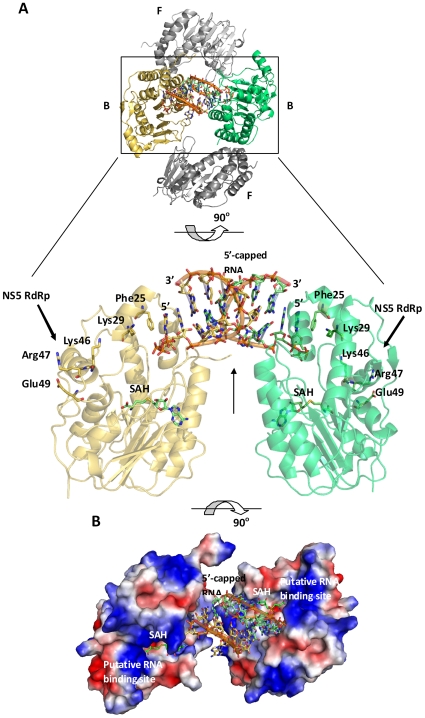
Structure of MTase in complex with 5′-capped RNA. (**A**) Cartoon representation of the four MTase monomers that surround the two RNA octamers. Two MTase proteins (in yellow and green) are bound (labelled “B”) to the RNA substrates whilst the other two monomers (in orange and grey) remain free (labelled “F”). The inset, a 90° rotation of panel A, shows a magnified view of the two MTase monomers that make contact with the two RNA monomers. These are related by a two-fold ncs axis perpendicular to the plane of the figure (top panel) and within the plane of the figure (bottom panel, represented by a vertical arrow). The putative interaction sites between the RdRp domain of NS5 and the MTase domain that was crystallized are indicated with arrows. (**B**) Electrostatic surface representation of the MTase-5′-capped RNA octamer (shown as sticks) complex. The co-purified SAH molecules are represented as sticks and the basic patch thought to be the catalytic relevant RNA binding site for methyl transfer is also labelled. Positively charged residues are in blue, negatively charged in red and non-charged residues in white. The view in panel B is the same as the top view of panel A with the two free MTase monomers removed for clarity.

Data collection and refinement statistics for the MTase from DENV-3 in complex with the 5′-capped RNA octamer are presented in Tables 1 and 2. The structure was refined to R = 0.205, R_free_ = 0.231 at a resolution of 2.90 Å. Clear electron density was visible for protein residues up to amino-acid 263 (the C-terminal end of the MTase construct is 272), for the two capped RNA octamers within the asymmetric unit (a.s.u) and also for the SAH by-products that co-purifies with the enzyme. This allowed building of all sixteen RNA bases (Figs. 1A and B). At the RNA 3′-end, the biotin-triethylene glycol (TEG; the 3′-end of the RNA was biotinylated) moiety is not visible, probably due to its flexibility. Four MTase monomers assemble into a ring structure that encircles the two RNA octamers around a 2-fold non-crystallographic symmetry (ncs) axis, whilst the other two monomers make no contact with RNA, but participate in protein-protein interactions (Fig. 2A). A view of the electrostatic surface of the two MTase monomers in complex with the capped octameric RNA dimer is shown in Fig. 2B. The basic putative RNA binding groove remains largely empty in our structure. Two sets of restraints were defined by restraining together the structures of the two RNA-bound monomers on one hand and the two free monomers on the other hand during refinement. In spite of the modest resolution, this procedure returned slightly improved R and Rfree values as compared to using a single set of restraints for all four Mtase monomers. Both RNA-bound MTase monomers (labelled “B” in Fig. 2A) are structurally very similar as are the two free MTase monomers (labelled “F” in Fig. 2A) with an average rmsd of 0.01 Å. By contrast, the average rmsd value between bound “B” and free “F” monomers is about 0.23 Å. Structural changes upon complex formation are located at the GTP binding pocket with amino-acid residues 21 to 23 displaced by distances of about 0.8 Å (Fig. 1A). A structural comparison of the bound MTase monomer (“B”) with the apo-DENV-2 MTase (PDB code 1L9K) [6] returns a rmsd value of 0.44 Å. Likewise, residues 21 to 23 at the GTP binding pocket are displaced by values between 0.9 to 1.7 Å. This further confirms that the structural changes observed for residues 21–23 of the MTase are induced upon RNA binding (Fig 1A), allowing neighbouring residues from the protein to establish contacts with RNA (listed in Table 3).

**Table 1:** Data collection statistics.

Parameters	5′-capped RNA
Space group	P3_2_
Cell parameters	
a,b,c (Å)	a = b = 137.5, c = 109.4
Resolution range a (Å)	60.0 – 2.9 (3.0 – 2.9)
Observed reflections	168,486 (24,557)
Unique reflections	50,667 (7,459)
Completeness (%)	99.1 (99.7)
Multiplicity	3.3 (3.3)
*R_merge_* b	0.098 (0.570)
Mean I/σ(I)	9.6 (2.2)
Monomers in a.u.	4
Ligands in a.u.	
5′-capped RNA	2
SAH	4

a The numbers in parentheses refer to the last (highest) resolution shell.

b *R_merge_* = Σ_h_Σ_i_|I_hi_-<I_h_>|/Σ_h,i_ I_hi_, where I_hi_ is the *i*th observation of the reflection h, while <I_h_> is its mean intensity. Abbreviation: a.u., asymmetric unit.

**Table 2:** Refinement statistics.

Parameters	5′-capped RNA			
Resolution range (Å)	49.7 – 2.9 (3.0 – 2.9)			
R_factor_ #,∧ (%)	20.5 (32.8)			
R_free_ * ^∧,^ (%)	23.1 (34.4)			
No of non-H atoms/chain	A	B	C	D
Protein	2075	2056	2060	2060
SAH	26	26	26	26
5′-capped RNA	204	204		
Glycerol	12			
Water	463			
Mean B-factor+ (Å^2^)				
Protein	20.1 (47.9)	20.0 (47.8)	20.0 (48.3)	20.0 (48.3)
SAH	59.8 (53.5)	56.8 (50.9)	54.7 (53.0)	55.0 (53.5)
5′-capped RNA	7.9 (87.1)	22.8 (87.0)		
Glycerol	85.1 (78.0)			
Water	26.7 (56.9)			
Rms deviations				
Bond lengths (Å)	0.0062			
Bond angles (°)	1.21			
Ramanchandran plot				
Most favoured (%)	92.8			
Allowed regions (%)	6.7			
Disallowed regions (%)	0.5			
Overall G factor$	0.15			

# R_factor_ = Σ ||F_obs_| - |F_calc_||/Σ | F_obs_|.

*R_free_ was calculated with 5% of reflections excluded from the whole refinement procedure.

$ G factor is an overall measure of structure quality from PROCHECK [41]. The numbers in parentheses refers to the last (highest) resolution shell.

∧ Bound (“B”) monomers and free (“F”) monomers were tightly restrained as two separate groups.

+ Mean B-factors are shown after TLS refinement. Mean B-factors without TLS refinement are given in parenthesis for comparison.

**Table 3:** Interactions observed between protein and RNA and within RNA.

Residue/Base* or Base/Base*	Between atoms	Bond type	Distance (Å)
Lys14/G_0_	NZ/2′-OH	Hydrogen bond	3.3/3.2
Leu17/G_0_	O/N2	Hydrogen bond	3.0/2.9
Asn18/A_1_	O/N1	Hydrogen bond	2.9/2.7
Asn18/G_0_	OD/2′OH	Hydrogen bond	2.6/2.7
Leu20/G_0_	O/N2	Hydrogen bond	2.4/2.5
Phe25/G_0_	Aromatic ring	Aromatic stacking	-
Pro152/A_1_	Pro152 ring and A_1_ ribose	Van der Waals	3.6/3.7
G_2_/G_8_	O6/N2	Hydrogen bond	2.7
G_2_/G_8_	N7/N1	Hydrogen bond	2.9
G_2_/A_4′_	O2/N1	Hydrogen bond	3.3
A_4_/U_7′_	N6/O4	Hydrogen bond	2.4

* Interactions between the MTase and the RNA octamer. Also listed the intramolecular RNA interactions. Primed numbers indicate residues related by the 2-fold ncs axis.

### Protein-RNA interactions

The interactions between the DENV-3 MTase and the 5′-capped RNA octamer are depicted in Figs. 1C, D and 2A. For simplicity, the 5′-capped RNA (“RNA I” in Fig. 1B) is labelled from G_0_ to G_8_ (5′-G_0ppp_A_1_G_2_A_3_A_4_C_5_C_6_U_7_G_8_-3′) and the RNA molecule related by the 2-fold ncs axis, (“RNA II”) from G_0′_ to G_8′_. The cap moiety occupies the GTP binding pocket while the remaining of the RNA chain makes minimal interactions with the protein and appears stabilized by intra-molecular interactions as described in the next section. Base G_0_ is sandwiched between Phe25 of the MTase GTP binding site and base A_1_. In addition, residues Lys14, Leu17, Leu20 and Asn18 together with several water molecules make interactions with the G_0ppp_A_1_ moiety (Table 3). Briefly, main chain interactions between the backbone carbonyl and amide groups of Leu17 and Leu20, respectively, allow hydrogen-bond formation with the C2 amine of residue G_0_. Residue Asn18 provides several contacts with both G_0_ and A_1_ including a hydrogen bond with the 2′-*O* of G_0_ through its carbonyl side chain, and with the carbonyl group of Pro152 through its amide side chain, positioning the Pro ring in the vicinity of A_1_ (Fig. 1C). These interactions are similar to those observed in other flaviviral structures bound to short cap analogues [20], [25]. The 3′-OH group of the A_1_ ribose points toward the protein in a “S1-like conformation” using the nomenclature defined in [20]. Such a conformation was proposed to obstruct the path for longer RNA chain, due to Pro152 and its surrounding residues. Interestingly, our structure shows that despite adopting a similar conformation, the path of the RNA chain at the 3′-OH of A_1_ is not affected by Pro152. Instead, Pro152 interacts with the ribose of A_1_ through a van der Waals interaction (Table 3). Water molecule mediated hydrogen bonds are observed between the hydroxyl group of Ser213 and the α-phosphate of the tri-phosphate linker. Another water molecule bridges the C6 amine of A_1_ and 2′-*O* of G_8_. Of note, the tri-phosphate group that connects the terminal guanine G_0_ with the rest of the RNA chain through a 5′-5′ linkage, adopts a U-shape (Fig. 1C). Interestingly, negative density (at a level of -3.5 σ) is found at this 5′-5′ linkage (but not on other phosphodiester linkages) suggesting flexibility of the tri-phosphate linkage of the RNA between G_0_ and A_1_. This flexibility might be relevant for the enzymatic function as it could facilitate repositioning of the RNA next to the SAM methyl donor during successive methylation events (see Discussion). Since neither the N7 nor 2′-O position of the RNA cap is positioned next to the methyl donor, the current structure does not represent a conformation competent for methylation.

The MTase domain was recently suggested to be endowed with the GTase activity [18], [19] **.** The GTase transfers the GMP moiety of GTP in a two–step reaction [26]. The first step involves the formation of a covalent guanylate-enzyme intermediate (GMP-E), where GMP is linked through a phosphoamide bound to a Lys residue of the enzyme. The second step involves the transfer of the GMP from the GMP-E complex to the 5′ diphosphate RNA (ppRNA), generating GpppRNA. For DENV NS5, Lys29 (conserved among all four serotypes of DENV) was suggested to be directly involved in the covalent attachment to the GMP [18], [19]. Mutation of Lys29 to Ala significantly reduced the efficiency of GTM-enzyme intermediate formation [19]. In our co-crystal structure, Lys29 is about 8 Å away from the α-phosphate of the cap structure. Overall, the structure may represent the product of guanylyation of the viral genome prior to the methylation events.

### Structure of the RNA octamers

**Figure 3:**
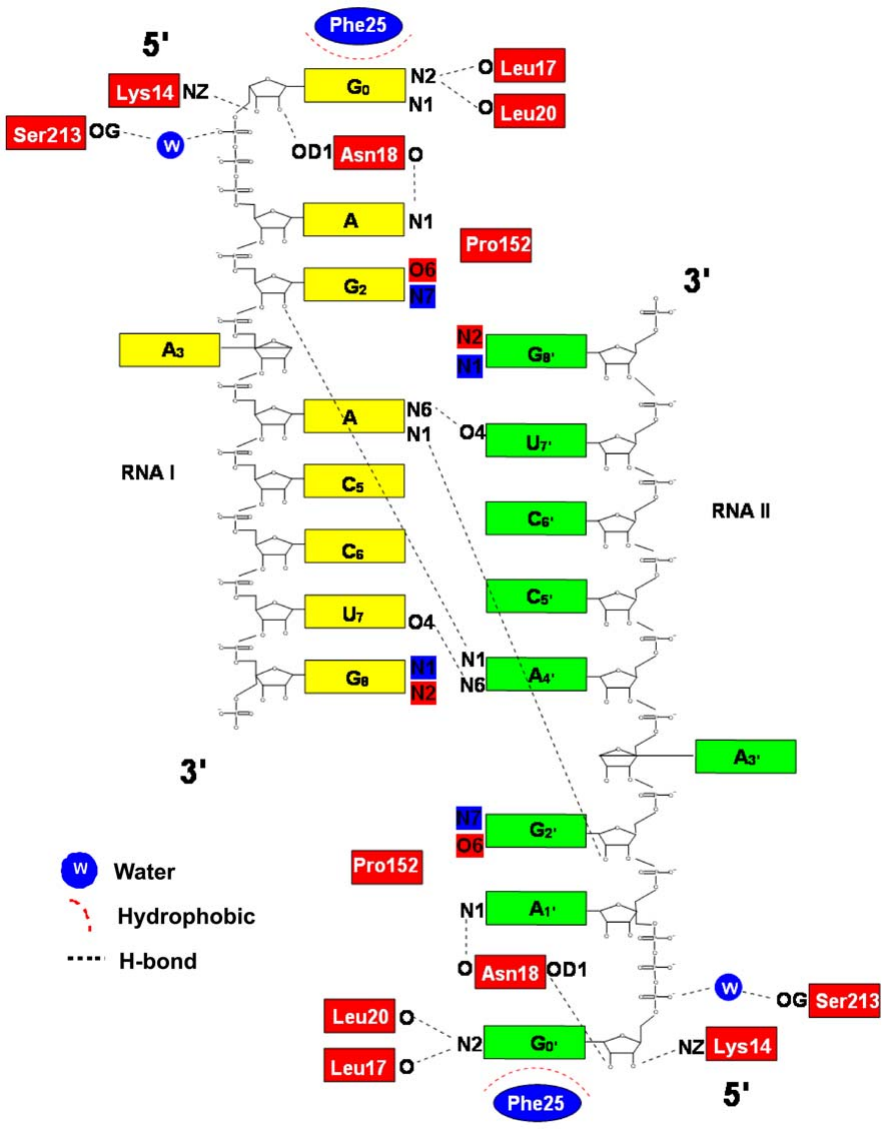
Schematic representation of the interactions between the two MTase monomers and the two RNA octamers. The two non-canonical base-pairs occurring within an RNA strand between G_2_-N7 and G_8_-N1/G_2_-O6 and G_8_-N2 are highlighted in blue and red respectively.

No direct interactions are formed between the MTase and the rest of the RNA chain: G_2_-G_8_. The RNA octamers contain no complementary sequences and stabilizing interactions between RNA molecules I and II arise through the formation of intra and intermolecular base stackings and non-canonical base-pairs, resulting in the formation of ‘kissing’ loops (Figs. 1B and 1D, and Fig. 3). Within each RNA octameric loop, six bases are stacked on top of one another. Thus, this RNA tertiary structure appears energetically stable, even in the absence of protein stabilizing factors. Each RNA turn consists of six bases, with adenine A_3_ flipping-out towards the solvent (Fig. 3). Eight non-canonical base-pairs are observed between the two RNA molecules related by the 2-fold ncs axis. Two sets of interactions (repeated twice by virtue of the 2-fold ncs axis that relate RNA I with RNA II) appear to play a key role in stabilizing the RNA structure: (1) Hoogsteen base-pairing between adenine A_4_ and uracil U_7′_ with the formation of two hydrogen bonds; (2) Guanine-guanine N7-N1 carbonyl-amino contacts between G_2_ and G_8_ with the formation of two hydrogen bonds. The complete list of interactions between both RNA chains (labeled RNA I and RNA II) is listed in Table 3. Of note, divalent metal ions (Mg^2+^ and Mn^2+^) that are key players in stabilizing RNA structures [27] were not included in our crystallization buffer which contained only Li^+^ and Na^+^ ions.

## Discussion

**Figure 4:**
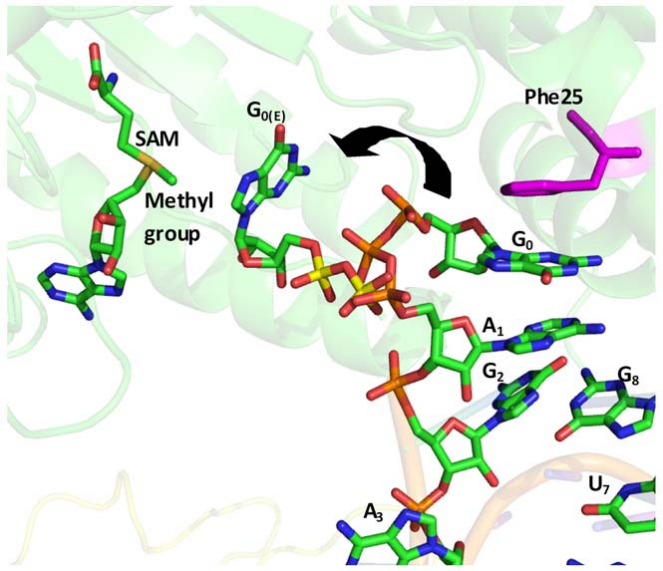
A hypothetical model for N7 methylation of G_0_ based on the observed interactions at the GTP binding site and the SAH molecule in the crystal structure. Extension of the tri-phosphate linker can bring the G_0_ moiety next to the methyl group of SAM to perform N7 methylation. The extended G_0_ is labelled as G_0(E)_. To distinguish the different tri-phosphate linkers, the phosphorus atoms of the di-phosphate groups of the extended form are coloured in yellow.

We report a crystal structure for the flavivirus MTase in complex with a 5′-capped octameric RNA. The structure reveals that the RNA substrate does not extend into the putative basic RNA binding cleft, as expected. Instead, the G_0ppp_A_1_ moiety of the RNA binds to the GTP binding pocket with the 3′-OH of A_1_ facing the protein, in the “S1 conformation” observed by using a short cap analogue [20]. This conformation was proposed to be an artefact since the 3′-OH of A_1_ was suggested to be incompatible with a longer RNA chain, due to obstruction by Pro152 and the surrounding residues. Interestingly, our structure shows that despite adopting a similar “S1-like” conformation, the continuation of the RNA chain at the 3′-OH of A_1_ is not affected by Pro152 and the surrounding residues. Instead, the bases and riboses of A_1_ and G_2_ form interactions with Pro152. As seen for short capped RNA analogues[20], [25], the protein-RNA interactions are mainly limited to its GTP binding site through contacts with G_0_ and A_1_ moieties. This suggests that the conformation of the RNA octamer adopted here is not compatible with a catalytically productive interaction with the MTase RNA binding groove. Therefore, other -probably longer- RNA chains mimicking the stem-loop architecture that is conserved at the 5′end of the flaviviral RNA genome are required to trap a complex fully relevant to methyl transfer. The atomic distance between the methyl donor in the SAM binding pocket and the N7 acceptor of G_0_ is about 16 Å in our structure. Therefore, it is conceivable that during the N7 methylation, the extension of the triphosphate linker will bring the G_0ppp_ moiety (more than 14 Å in length) in the immediate vicinity of the methyl group of SAM, allowing methyl transfer (Fig. 4). Based on the mutagenesis results of the WNV MTase, it was proposed that two distinct sets of amino acids on the enzyme surface (including residues in the RNA binding site) are required to reposition the RNA cap during the N7 and 2′-*O* methylation events [24], [28]. Furthermore, mutagenesis results of WNV RNA substrate indicate that distinct viral RNA elements are required for the two methylation reactions [24], [29]. At this point, it therefore remains difficult to precisely visualize the RNA-protein interactions during and after the two methylation events.

Our structure does not represent a catalytically-competent complex for either the N7 or 2′-*O* methylation. However, the observation of a RNA dimer surrounded by four MTase monomers in our crystal structure suggests some intriguing possibilities of possible biological relevance: The RNA dimer is bound to two MTase monomers, whilst the two other monomers do not have direct interactions with the RNA, and each of the four monomers contain the ligand SAH (Fig. 2A). One possibility is that this crystal structure mimics the initial docking of the 5′-capped viral genomic RNA to the MTase at the GTP binding site prior to the N7 and 2′-*O* methylation events. This is likely to follow the GTase reaction proposed [20] whereby the GTP and the nascent viral RNA, _pp_AG-RNA (generated by the NS3 5′-RNA triphosphatase to remove the 5′-γ-phosphate) is enzymatically linked with the release of pyrophosphate. Subsequently, conformational rearrangements must occur in order to bring the viral genomic RNA substrate in close proximity to the methyl donor to allow methyl transfer. Results from the PISA server [30] suggest that the quaternary assembly observed in the present crystal form is not stable in solution (Supp. Table S1). However, in the context of infected cells within the membrane-bound RNA replication complex, comparable quaternary assemblies might be formed. Thus, another attractive possibility is that several MTase monomers might cooperate together with the NS3 protein, previously demonstrated to interact with NS5 [31], [32]. Indeed, a ring-like structure of MTase monomers encircling a RNA substrate, as observed here would allow the 5′-capped RNA to sequentially dissociate from one MTase and bind to another MTase to perform the various methylation events needed to complete cap formation of the viral genome. Thus, our crystal structure may provide a structural explanation for how distinct mutant MTases defective in N7 or 2′-*O* methylation could trans-complement one another, resulting in double methylated ^7me^GpppA_2′-*O*-me_ product [33]. This is reminiscent of the reovirus λ2 protein-mediated RNA cap formation, in which the GTase, N7 methylation, and 2′-*O* methylation are sequentially executed by separate domains [34]. A similar hypothesis was put forward by Assenberg et al. based on the crystal structure of the Murray Valley encephalitis virus (MVEV) NS5 MTase domain complexed with G_ppp_A cap analogue (see PDB 2PXC [35]). In this work, the authors proposed that two MTase monomers would cooperate to sequentially methylate viral RNA trapped at the dimeric interface. A comparison of the quaternary structures adopted by the NS5 MTase from MVEV and the DENV MTase (this work) as well as the path taken by the bound nucleic acid in each case is shown in Figures S1, S2 and S3. This comparison shows partial overlap between the two structures in the way they encircle bound RNA (Fig. S2) but the exact path of the RNA moiety is clearly different (Figs. S1 and S3).

Based on a reverse genetic analysis of the DENV-2 MTase and the RdRp domains, complemented by *in silico* docking approach to map their putative interactions, a model for the full-length WNV NS5 protein was put forward [36]. This model identifies interactions between Lys46, Arg47 and Glu49 of the MTase with Leu512 of the RdRp (Fig. 2A). Of note, Lys29, a residue proposed to be involved in GTase activity [18], [19], [25], lies in the vicinity of this putative interface (Fig. 2A, inset). One possibility is that the nascent viral ppp-A-RNA emerging from the RdRp domain binds to NS3 to remove the 5′-γ-phosphate; the resulting ppA-RNA is capped by the MTase domain prior to the formation of replication complex in which both the NS3 and/or NS5 proteins are expected to interact [18], [32], [37]. In conclusion, the supra-molecular arrangement observed in the crystal structure points to a specific mode of recognition by the NS5 protein of evolutionarily conserved and functionally important RNA tertiary structures that are known to be located at the 5′ or 3′ end of the viral genome. Such RNA structures are likely to nucleate the assembly of several non-structural viral and cellular proteins for the initial formation of a membrane-bound RNA replication complex. Thus, further work mapping the interactions between the NS3 and NS5 proteins with evolutionarily conserved viral RNA stem-loop structures located at the 5′ and 3′ ends of the viral genome, as well as the time sequence of their interactions is needed to answer these questions.

## Materials and Methods

### Expression and purification of DENV-3 MTase

*E. coli* BL-21 (RIL) competent cells were transformed with pGEX4T-1 DENV-3 MTase (1-272 amino acids and grown in LB plates containing 100 µg/mL ampicillin and 50 µg/mL chloramphenicol at 37°C overnight. A single colony was picked and inoculated into 5 mL LB medium containing 100 µg/mL ampicillin and 50 µg/mL chloramphenicol and grown at 37°C overnight. The overnight cultures were then transferred to 500 mL fresh LB media and the cultures were incubated at 37°C with shaking (220 rpm) to an OD_595_ between 0.6 to 0.8. Protein expression was induced by adding isopropyl-β-D-thiogalactopyranoside (IPTG) to a final concentration of 0.4 mM. The cultures were incubated overnight with shaking (220 rpm) at 16°C. Cells were then harvested by centrifugation at 6000 rpm for 10–15 minutes at 4°C. Cells were then lyzed by sonication for 30 minutes followed by centrifugation at 20 500 rpm for one hour. The protein supernatant was then purified using glutathione S-transferase (GST prep FF16/10) affinity column. The column was washed with buffer A (20 mM Tris-HCl pH 7.5, 200 mM NaCl, 2 mM β-ME and 10% glycerol) for five column volumes after sample injection. Proteins were eluted with a linear concentration gradient of reduced glutathione (GSH) from 0 to 10 mM. The collected fractions were dialyzed in buffer A to remove GSH, with the concomitant cleavage of the thioredoxin tag by thrombin digestion at 4°C overnight. The protein was then further purified by gel filtration using HiLoad 16/60 Superdex 75 column that was pre-equilibrated with 20 mM Tris-HCl pH 7.5, 200 mM NaCl, 2 mM DTT and 10% glycerol. Collected fractions containing pure MTase were pooled and concentrated to 16 mg/ml before storing at −80°C.

### Crystallization and Data collection

To obtain crystals of the binary complex between MTase and 5′-capped RNA, the biotinylated 5′-capped RNA octamer with the sequence 5′-G_ppp_AGAACCUG-biotin-TEG-3′ [23] was co-crystallized with 4 mg/mL of DENV-3 MTase with a molar ratio of 1.7∶1 using the hanging drop vapour diffusion method. A volume of 2 µL of reservoir solution containing 20% PEG 4000, 0.4 M Li_2_SO_4_ and 0.1 M sodium-citrate pH 5.0, was mixed with an equal volume of MTase-RNA solution. Small, hexagonally shaped crystals were observed after incubation at 18°C for one week. For data collection, crystals were transferred to its mother liquor supplemented with 20% (v/v) glycerol as cryo-protectant before being mounted and froze in liquid nitrogen. Diffraction experiments were performed at the PXII (X10SA) beam line at the Swiss Light Source, Paul Scherrer Institut, Villigen, Switzerland. Indexing, integration, scaling and merging of intensities were carried out using MOSFLM [38] and SCALA from CCP4i package [39]. The crystals parameters and data collection statistics are summarized in Table 1.

### Structure determination and refinement

All structures were determined by molecular replacement using the MOLREP program from the CCP4i package using DENV-2 MTase (PDB code: 2P3O) as search probe. Structure refinement was carried out using REFMAC5 with TLS refinement [39], with each chain being defined as a separate group. Manual model rebuilding between refinement cycles was performed using Coot [40]. The quality of the structures was analyzed using PROCHECK [41]. Figures were prepared using Pymol [42]. Refinement statistics and stereochemistry analyses are summarized in Table 2.

### Protein Data Bank accession code

The atomic coordinates and structure factors have been deposited in the Protein Data Bank with accession code 2XBM.

## Supporting Information

Table S1 Buried surface areas. To assess the stability of the quaternary assembly of MTase molecules "A, B, C, D", we calculated buried interfaces. Monomer A is bound to F and monomer B to E. We note rather small interfaces for protein-protein interactions (by comparison stable antigen-antibody interfaces bury at least 1200 Å2), but a rather large RNA-RNA interface of 405 Å2 (RNA molecules are labeled "E" and "F"). Thus RNA would play a major role in stabilizing such a quaternary assembly in the virus replication complex.
(0.05 MB DOC)

Figure S1 Comparison of the paths taken by the RNA fragments in our structure and in structure 2PXC. One Mtase monomer (yellow) of the crystallographic dimer from 2pxc is superimposed with one bound MTase monomer A of our structure (green). The Gp moieties at the 5′ end are superimposable. (RNA is green for our structure and in yellow for cap analogue). From the β-phosphate onwards, the RNA structure forms a loop and protrudes out of the protein in our structure.
(0.46 MB TIF)

Figure S2 After monomer superposition (in green and yellow), the other monomer (pink) of the crystallographic dimer from structure 2pxc has a ∼60 degrees difference in orientation compared to the nearest neighbour of DENV MTase monomer D (green).
(0.48 MB TIF)

Figure S3 Superposition of the crystallographic dimer of 2pxc with DEN MTase monomers A and B (both bound to RNA).
(0.52 MB TIF)
