# Supplementary material for: Crystal Structure of the Dengue Virus Methyltransferase Bound to a 5′-Capped Octameric RNA
Source: PLoS One. 2010 Sep 17;5(9):e12836. doi: 10.1371/journal.pone.0012836 (PMC2941465; doi:10.1371/journal.pone.0012836)
Supplement: Table S1 — Buried surface areas. To assess the stability of the quaternary assembly of MTase molecules "A, B, C, D", we calculated buried interfaces. Monomer A is bound to F and monomer B to E. We note rather small interfaces for protein-protein interactions (by comparison stable antigen-antibody interfaces bury at least 1200 Å2), but a rather large RNA-RNA interface of 405 Å2 (RNA molecules are labeled "E" and "F"). Thus RNA would play a major role in stabilizing such a quaternary assembly in the virus replication complex. (0.05 MB DOC) [file pone.0012836.s001.doc]

**Supplementary Table 1: Buried surface areas**

To assess the stability of the quaternary assembly of Mtase molecules “A, B, C, D”, we calculated buried interfaces. Monomer A is bound to F and monomer B to E. We note rather small interfaces for protein-protein interactions (by comparison stable antigen-antibody interfaces bury at least 1200 Å^2^), but a rather large RNA-RNA interface of 405 Å^2^ (RNA molecules are labeled “E” and “F”). Thus RNA would play a major role in stabilizing such a quaternary assembly in the virus replication complex.

| Monomers | Interface area (Å^2^) |
| --- | --- |
| A/C | 760 |
| B/D | 610 |
| B/C | 275 |
| A/D | 275 |
| A/F | 185 |
| B/E | 175 |
| E/F | 405 |
