# Supplementary figures and images for: Crystal Structure of the Dengue Virus Methyltransferase Bound to a 5′-Capped Octameric RNA
Source: PLoS One. 2010 Sep 17;5(9):e12836. doi: 10.1371/journal.pone.0012836 (PMC2941465; doi:10.1371/journal.pone.0012836)

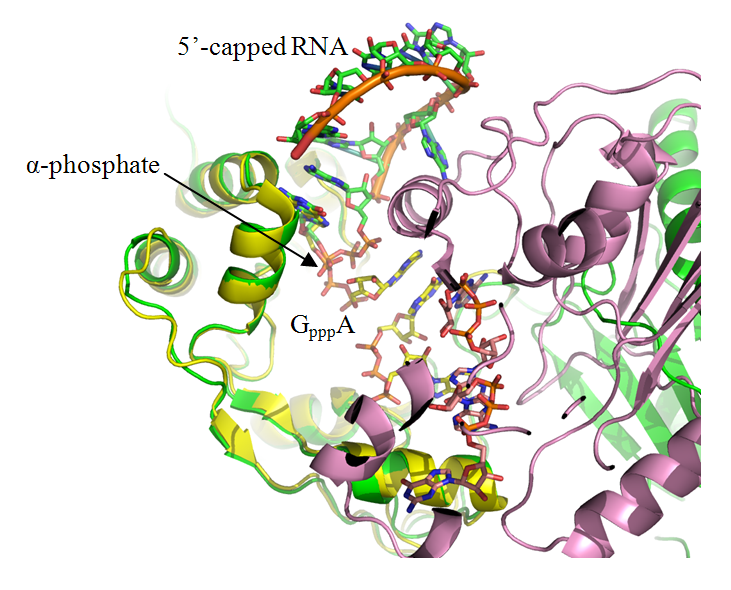

Supplement: Figure S1 — Comparison of the paths taken by the RNA fragments in our structure and in structure 2PXC. One Mtase monomer (yellow) of the crystallographic dimer from 2pxc is superimposed with one bound MTase monomer A of our structure (green). The Gp moieties at the 5′ end are superimposable. (RNA is green for our structure and in yellow for cap analogue). From the β-phosphate onwards, the RNA structure forms a loop and protrudes out of the protein in our structure. (0.46 MB TIF) [file pone.0012836.s002.tif]

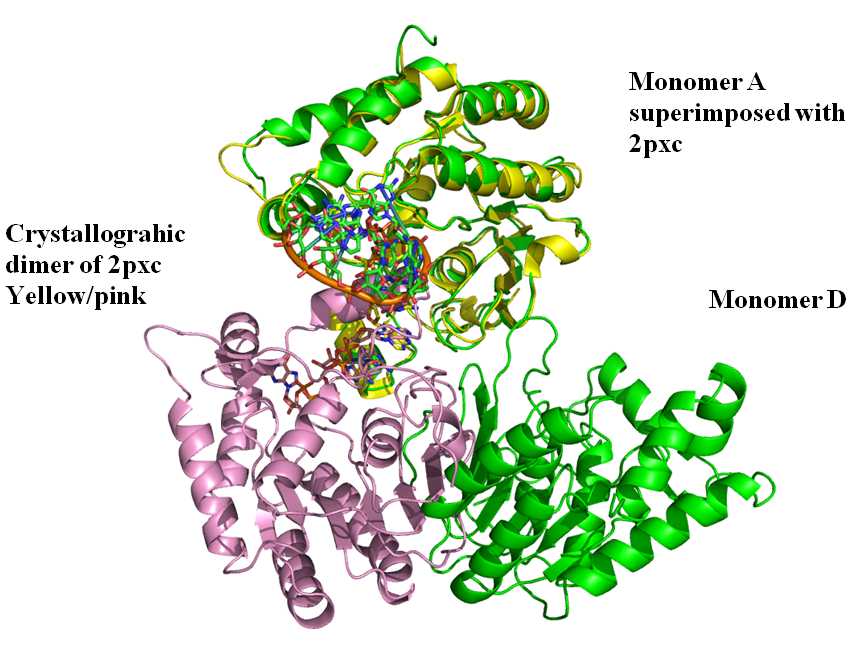

Supplement: Figure S2 — After monomer superposition (in green and yellow), the other monomer (pink) of the crystallographic dimer from structure 2pxc has a ∼60 degrees difference in orientation compared to the nearest neighbour of DENV MTase monomer D (green). (0.48 MB TIF) [file pone.0012836.s003.tif]

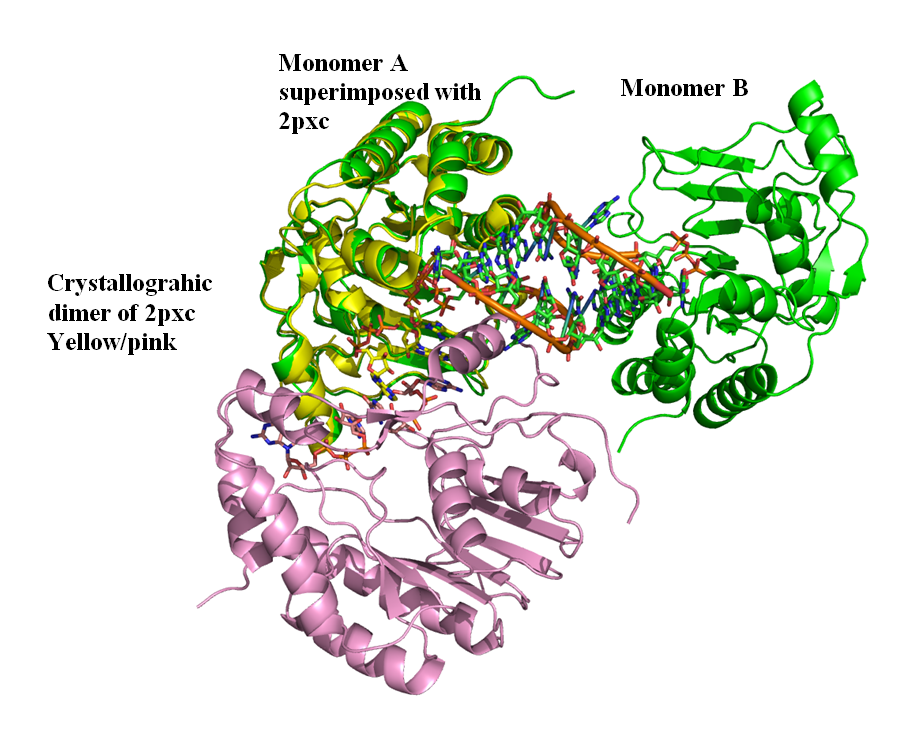

Supplement: Figure S3 — Superposition of the crystallographic dimer of 2pxc with DEN MTase monomers A and B (both bound to RNA). (0.52 MB TIF) [file pone.0012836.s004.tif]
